# Commentaries on the Causes Forms, Symptoms, and Treatment of Insanity

**Published:** 1829-01-01

**Authors:** 


### Medico-Chirurgical Review,

### No. XIX.

OCTOBER 1, 1828, TO JANUARY 1, 1829.

### I.

### INSANITY.

COMMENTARIES ON THE CAUSES, FORMS, SYMPTOMS, AND TREATMENT, MORAL AND MEDICAL, OF INSANITY. By *George Man Burrows,* M.D. &c. &c. 8vo, pp. 716. Underwood, 1828.

IT was high time that England should produce a work on INSANITY which would concentrate the lights that have been thrown on that dreadful malady by various pathologists and practitioners, during the last twenty years. Till the appearance of the work at the head of this article, there was no systematic treatise on the subject of mental derangement, in the English language, which was at all on a level with the progress of pathology in Europe, and therefore Dr. Burrows' attempt to supply the deficiency is meritorious, and the result will be beneficial to the profession in this country. Insanity is a disease so obscure in its nature, and so untoward in its manifestations, that the great mass of medical practitioners consider it as one of those which ought to go at once to the asylum, and, therefore, they do not study it with that care which is expended on the common diseases of routine practice. This circumstance may have contributed to retard the progress of mental pathology in this country—for, it is not to be concealed that medicine, in all its branches, and in this one particularly, is become a *trade* as well as a science here. How few professed oculists, aurists, or dentists, *publish* on the subjects of their especial pursuits—except in the Newspapers, or in works where the *means* are concealed, though the *end* occasionally becomes manifest! !* What information, on the interesting subject of insanity, have we derived from the accumulated experience of the numerous physicians and surgeons in these islands, who devote their time and talents *exclusively* to this melancholy class of afflictions? Dr. Haslam, on resigning the seals (or at least the *scales,*) of Bedlam, favoured the world with some observations on sound and unsound mind; but there was rather too much of philosophy and metaphysics mixed up with the Doctor's practical remarks,

* By the expression " few," we admit some honorable exceptions to the general rule—a rule which applies still more strongly to the treatment of insanity than to the treatment of any other class of affections.

to render them as useful as they might have been, considering the field which had been open to the author for investigation. Since Dr. Haslam's book appeared, there has been nothing published in this country which deserves particular notice, although many valuable monographs issued from the press on the Continent, during that period. Pinel, Esquirol, Georget, and many other writers, have greatly enriched this field of medical science, which has lain nearly waste in the land where insanity is said to thrive better than in any other region of the world.

It is well known that Dr. Burrows, more than ten years ago, issued proposals for the publication of a large work on Insanity. We know not whether to congratulate or condole with the author, on an accident which happened to his manuscripts when nearly ready for the press. By one "COUP DE MAIN" of a thief, the whole of his literary researches were swept away! We should have liked to see the PHRENZY of the SPOLIATOR, on opening the large desk, with which he leisurely marched out of the front door of the Doctor's house, in open day. Instead of BANK-NOTES, he only found "NOTES ON MADNESS!" To what purpose these notes were afterwards applied, has never been ascertained:—But we verily believe that both the author and the public will be benefited by the operations of the thief. Learning may have lost—but knowledge has been gained by the accident. Dr. B. could have had but little personal experience, ten years ago, as compared with the present, in the science and treatment of insanity. The loss of his manuscripts has thrown him more on his own resources; and it would be well for MEDICINE if a thief were always in the way, when an author first steps out to have an interview with that important personage, the printer!

Dr. Burrows has divided his work into five parts, and each of these parts are subdivided into commentaries. We can see no real necessity for the division into PARTS, because there are no natural distinctions between them—the commentaries being quite sufficient for localising the various points of discussion.

THE FIRST PART, occupying 245 pages of letter-press, and embracing the important subject of ETIOLOGY in all its various bearings, together with commentaries on the states of the nervous and vascular systems—hæmorrhages, complications of insanity with other diseases, metastases, consequences, &c. would, in strict justice, demand an entire article in this Journal. Fifteen years exercise has not opened to us the ROYAL ROAD in REVIEWING. We cannot despatch a large volume in two or three pages according to the modern "MARCH OF INTELLECT," which indeed is more like the aerial flight of a balloon than the sober pace of literature or science. In these *gazeous* excursions, the land may be distinguished from the water—the mountain from the valley—or perhaps the city from the country; but all other and minute features of the scene are veiled from the sight. It is so with MODERN REVIEWING. Instead of exhibiting the minute features of a work, not even the leading characters are portrayed. In short,—to eulogise or condemn a publication, unread—if not unseen—is no uncommon practice in these days of venality, licentiousness, and personal rancour.

We shall not dwell on the "INTRODUCTION" to the work, nor enquire whether "madness is one of the curses imposed by the wrath of the Almighty on his people for their sins." Dr. B. we understand, has been censured for this expression, having forgotten to give his authority (Deuteronomy) for it, and consequently appearing to give it on his own. With all due respect for the Doctor, as well as for Deuteronomy, we do not believe that the Almighty ever afflicted one of our fellow-creatures with madness, the Heathen authority to the contrary notwithstanding:—

Quem DEUS vult perdere prius DEMENTIT.

The antiquity of madness is pretty high, since SAUL was in that condition, and appears to have been cured of melancholia by the music of David's harp. That the disease had multiplied between the days of David and Horace is sufficiently evident from the satirical allusions ("insanire omnes") of the Roman Poet. But these matters must be all left on one side, while we proceed to more important considerations. After exposing the absurd attempts to investigate the nature of insanity as a purely *mental* disorder, Dr. B. quotes a passage from BACON, which shews that HE clearly saw the true path of investigation. Speaking of disorders of the mind, "the absolute source, if carefully developed (says he) will be found to exist in *corporeal changes*, or the effects of external agents acting on the *gross machine*, and not primarily on the immaterial principle, as has, unfortunately for the subjects of disease, been too commonly apprehended."* It is rather mortifying to think that, even in the year 1827, it was publickly maintained in some of our best medical societies of this metropolis—and by men who are charged with the instruction of medical youth—that insanity might exist as a purely mental disease, and quite independent of corporeal disorder!! This fact proves how ill understood is the subject of insanity, even among the better informed classes of the profession.

### COMMENTARY THE FIRST—MORAL CAUSES.

The causes of insanity are now pretty generally agreed to be moral and physical.

"Every impression on the sensorium, through the external senses, and every passion in excess, may become a moral cause of insanity. Thus all, however opposite, act as exciting causes, and will produce this result: joy and grief, anger and pain, love and hatred, courage and fear, temperance and ebriety, repletion and inanition, application and indolence, may have the same effect. Vices, also, which occasion changes in the physical constitution, act as remote moral causes, and induce mental derangement." 9.

All moral impressions affecting the feelings act first on the brain and nervous system—then on the heart and vascular system, which latter re-act on the former:—

"Hence there are two impressions: the one primitive, affecting the sensorium; the other, consecutive, but simultaneously affecting the heart. Thus the nervous and vascular systems are both implicated; and in this manner moral impressions become causes of insanity. The moral cause, therefore, is always the remote cause; the physical, *the proximate*, or that state of the cerebral functions which immediately precedes the peculiar action denominated maniacal." 10.

* Novum Organon.

It is easy to see—or, at least, to believe, that this play of sympathies, or action and re-action of the two systems, nervous and vascular, must disturb the functions of both—and even affect their structure if long continued or excessive in degree.

It appears that the upper classes of society are rather less subject to insanity than the lower. The *former* suffer more from the moral causes—the latter, from the *physical* causes of the disease. In respect to the indigent classes, Dr. B. avers that the majority of cases of insanity, in them, "originate in direct *physical* causes, which the privations, and consequent misery, the poor suffer in all countries, as well as their vices, greatly multiply." Dr. B. does not seem to have been able to connect insanity with trades or professions—but acknowledges, with Rush, Pinel, Hallaran, and others, the powerful influence of political or civil revolutions of states, in the production of mental alienation.

The question now presents itself—do these moral causes, say grief, terror, jealousy, &c. operate *directly* on the mind, and induce mental derangement? —or must there always be an intervening corporeal disorder?—We would incline to the latter position, and it is evident that Dr. Burrows is of a similar opinion.

"All emotions of the mind, it is evident, are capable of disturbing the corporeal functions; and though in themselves moral causes, they become physical in their operation. Hence physical causes grow out of moral causes, and these frequently lead to insanity; *not, however, by direct impressions on the organ of the mind, but through the means of those morbid changes in the system which they gradually effect.*" 22.

Thus, then, insanity is a corporeal disease, and the manifestations of the mind are disordered because the organ of the mind is disordered.

The following passage will probably excite some criticism.

"Could we imagine a human being void of all feeling, moral or religious, mental derangement is not there to be expected through a moral cause. But even where reason is wanting, instinct prevails; and brutes have their passions, which, when excited to excess, or thwarted, produce madness." 23.

If by madness Dr. B. merely means a temporary furor, (*ira* brevis furor est) then we agree that a horse or any other animal is liable to madness; but we question whether BRUTES are ever insane, in the common acceptation of the word. Monomania is the most frequent of all forms of insanity—and can Dr. B. prove that a brute has ever evinced insanity on a *single point*, being sane on all others?

In the second Commentary Dr. Burrows discusses the knotty point whether RELIGION should be looked upon as the cause or the consequence of insanity. Dr. B. properly remarks that any single passion, excited to excess, may induce mental derangement—and consequently RELIGION, "which influences the internal man more than the passions collectively, may be a cause of insanity."

"On the other hand, there is no doubt, that a lunatic may imbibe a religious as well as another hallucination, and yet be insane from a cause the reverse of religious. In the one case, however, it is a cause; in the other, an effect.

"Now a great source of error seems to arise from the confounding of this necessary distinction.

"Medical writers who have derived their chief experience from public practice, are most apt to err in this particular. The previous history of lunatics admitted into public asylums is rarely known; therefore the moral cause of the malady is frequently inferred from the tenour of their mental aberrations; than which nothing can be more deceptious. Hence it is to be feared, that many cases have been hastily attributed to a religious origin, merely because the conduct or conversation of the lunatic has exhibited traits of too vivid spiritual impressions. In private practice the opportunities of obtaining this essential information are superior; and upon a point of such serious importance, I have not omitted to avail myself of them." 25.

This is, perhaps, the true state of the case. We find a certain proportion of lunatics in an asylum harping on some religious topic, or fancying themselves deities of an upper or lower region. A hasty observer would attribute all these instances of monomania to religious meditation. It is probable, however, that one half of them were merely what is called "serious people," who being exposed to some of the various causes of insanity, exhibited, when insane, a religious hallucination. But as deep meditation on the mysteries of religion tends, in general, to perplex and bewilder the strongest intellect, we may easily conceive that an ignorant person, tinged perhaps with superstition, bigotry, or fanaticism, would readily become deranged in mind by lucubrations so unfitted for his intellects. Dr. Burrows appears to labour hard to shew that *pure christianity* never leads to madness—but, "that it is from the *perversion* of it that many become the victims of insanity." We conceive that this is "splitting hairs"—or rather, "cutting blocks with a razor." It is the same as proving that no man is ever injured by medicine, but only by the perverse administration thereof. Thus, a man takes too large a dose of emetic tartar, and dies. Dr. Burrows would say, t'was not the tartrite of antimony that killed the man, but the mal-administration of the remedy. But granting that it is the *perversion* of christianity which leads to insanity, who is to be the arbiter of this perversion?

"Who can direct, when all pretend to know."

We confess that we do not clearly comprehend the drift of the following passage—though we are ready to admit that the want of comprehension is attributable to our own astuteness of intellect.

"But, although this be admitted, there is not a tittle of evidence to substantiate, that Christianity, abstractedly, ever produced that effect. Such accusations are the abortions of infidelity, or of those who lack knowledge. Religion may have been reproached by careless observers as the source of mental derangement, because it is often associated with misery;—for affliction induces many earnestly to seek religious consolation, who previously never thought of it, or who but mechanically followed its outward forms. In minds broken down by adversity, and little acquainted with its genuine precepts, a consequence, opposite to that which was sought and expected from religion, sometimes ensues: in this case the moral feelings have greater force than the spiritual, and the disappointment is not the default of the principles of the Christian faith.

"Constitutional temperament also interposes, and often distorts the truth; and thus generates an opinion, that melancholic insanity is the effect of religious impressions. Minds so framed view all the blessings of this, or a future life, by involution. Their greatest gratification is persistive despondency. Deaf to precept or example, they retort:—

"Go—you may call it madness—folly—
You shall not chase my gloom away;
There's such a charm in melancholy—
I would not, if I could, be gay!"

But we shall leave this subject—convinced as we are, and even at the risk of being called infidels or ignorantes, that religious meditations, with the best intentions, precipitate many a mind, weak and strong, into the gulf of madness. This, indeed, is admitted plainly enough by Dr. B. himself at the end of the commentary.

"Were I to allege one cause which I thought was operating with more force than another to increase the victims of insanity, I should pronounce, that it was the overweening zeal with which it is attempted to impress on youth the subtle distinctions of theology, and an unrelenting devotion to a dubious doctrine." 56.

### COMMENT. III.—PHYSICAL CAUSES.

We now come to more tangible, but not less debateable ground. Dr. B. justly observes that "the great obstacle to the knowledge of the pathology of insanity, has been the long-prevailing error of studying the *mental* to the neglect of the corporeal phenomena which are *almost always cognizable*."

"The hallucinations of the mind being clearly only the signs of *its* disorder, as symptoms are of corporeal disorders, they are but of secondary importance in the study of insanity." 59.

Here Dr. B. falls into the usual error of attributing disease or disorder to the *mind*. If the mind be immaterial, it cannot be subject to disease. If material, then it is a part of the body, and the cause of disorder is still material. A patch of erysipelas on the skin, or a thorn in the foot, will cause delirium, in which the functions of the mind are as completely deranged, to appearance, as in raging insanity. Yet, who would say this is a disorder of the immortal mind? Is it not clearly a disorder of the body perverting the manifestations of the mind? We perfectly agree with Dr. B. that it is useless—worse than useless, to dwell on the *mental* aberrations. Of what consequence is it, whether the lunatic fancies himself a god or a devil—a philosopher or a conjuror—a tea-pot, or a tiger? "Ought we not to prefer examining the various signs which indicate *functional* or *structural* lesion? It is useless to go over again, with Dr. Burrows, the discrepancies between the material and immaterial doctrines of insanity. Dr. B. in commenting on Mr. Coombe, makes use of the following expression.

"Can any comparison be endured between the *divine immaterial function of the brain*, and the palpable and material functions of other parts of the human body." 65.

The *divine immaterial* functions of a mass of *matter*! The *palpable* functions of other parts of the body! Has Dr. B. ever seen or fingered the *function* of secretion, for example? We can see *blood* going to the liver, and we see *bile* coming from it;—but if any man ever saw the *function* of secretion, by which the one is converted into, or separated from, the other, we are ignorant of his existence. On the contrary, we fearlessly maintain that the secretion of bile in the liver is just as great a mystery to us, as the secretion of Paradise Lost from the brain of Milton.

In this country, Dr. Marshall appears to have been the first who conceived that insanity might be a corporeal disease, and dependent on disease of the brain. For this opinion he was violently assailed by the late John Hunter. To support his doctrine, Dr. M. made many dissections at the Old Bethlem; but his Morbid Anatomy of the Brain, in mania and hydrophobia, was not published till 1815, after the author's death. Haslam's dissections preceded (in point of publication) those of Marshall. Both tended to illustrate the subject of inquiry, "and fully prove a morbid condition in the encephalon of the maniacs submitted to their inspection."

"However, when it was proved, beyond dispute, that the brains of maniacs commonly presented morbid appearances, it was assumed that these alterations were the causes of the disorder; but it was soon objected, that such were the effects, and not the causes, of intellectual derangement. Among these sceptics were Crowther, Black, &c., and their doubts coincide with the opinion of many eminent physiologists who have since entered upon the inquiry. Others, while they cannot yield acquiescence in these conclusions, admit them with certain modifications." 69.

Bonetus, Morgagni, and other anatomists, indeed, had not been able *always* to find corporeal disorder in the heads of maniacs—and such is the case with modern pathologists.

"I have myself (says Dr. B.) assisted at several accurate anatomical investigations, conducted by eminent demonstrators, of the crania of insane patients who have been under my care, and who had exhibited up to the hour of their decease the most furious symptoms of mania for months, and yet not a vestige of disease could be traced." 70.

But, as Dr. B. properly observes, we are not in a condition to *know* when disease exists in the brain. Could any one *detect* the changes which obtain during functional disorders in the brain and other organs? No, indeed! And yet there must always be some change in a part corresponding with the functional disorder of that part, however incapable we may be of detecting it by the scalpel.

"This, however, is certain—that when an insane person has been cut off by an acute disease or accident, or has destroyed himself, and the insanity has been of short duration, there are seldom exhibited any alterations, or morbid appearances in the encephalon, beyond slight vascular congestions or effusion. But in long-standing cases, a *post-mortem* examination generally exhibits strong evidence of disease in that organ. Sometimes, however, violent insanity has continued for years, and not a visible trace of diseased structure or action has been discovered in the brain or elsewhere." 71.

Dr. B. asks, "is not the human body subject to, and influenced by peculiar diatheses?" He refers to the apoplectic, hydrocephalic, scrofulous, and gouty diatheses, which may exist without any appreciable change of structure.

"Were we to insulate a portion of a dead body, and examine its texture, without a previous knowledge of the disease affecting the person while living, could we decide what disease had existed? Why, therefore, should not the brain be influenced by the same laws, and undergo a specific change, and be the site of disease of which there is no visible trace? Why, in fine, should we expect to discover by the eye the maniacal diathesis, when all others are impenetrable?" 73.

Dr. B. asks farther:—"who can say that excitement, nay inflammation itself, has not existed in the brain, if the circulation through a great part of it is, as it is said to be, colourless?" A morbid action, as we before observed, of other important organs is indicated by appropriate symptoms; and yet, on dissection, the organ suspected often appears sound. It is so with the brain. It may be, and is, incapable of manifesting correctly the operations of the mind, long before its disorder is appreciable by the senses.

To argue, as some have done, that, as there is no appreciable lesion of brain in insanity, so there is no use in directing any material remedies to the body, is sheer nonsense. The same argument might equally be applied to all other diseases. They are at first functional, and the appreciable change is a subsequent process.

In respect to the changes actually found—and we believe they will be found in at least four cases out of five—after death, there is no doubt that some of them are *post mortem* changes, and therefore not to be considered either as causes or effects of insanity. Serous effusion may be instanced in illustration.

"Much important information may be elicited from the labours of men possessing such extensive opportunities as Greding, Esquirol, Georget, Neumann, &c. And the collection made by Dr. Scipion Pinel, embracing those of Messrs. Esquirol, Villermai, Beauvais, and Schwilgaé, and which, in the aggregate, comprise two hundred and fifty-nine dissections of maniacs, offers a mass of pathological examinations particularly worthy of record. They are thus classed:—

| | | |
|---|---|---|
| "*Lesions of the Brain.* | | |
| "Apoplexy | 27 | |
| Organic lesion of the substance of the brain | 19 | |
| Organic chronic lesion of the membranes | 22 | |
| | — | 68 |
| "*Lesions of other Organs.* | | |
| "Chronic peripneumony | 20 | |
| Phthisis | 22 | |
| Chronic peritonitis | 9 | |
| Chronic pleuritis | 7 | |
| Chronic inflammation of the digestive canal | 50 | |
| Other organic lesions of this viscus | 13 | |
| Lesions of the liver | 5 | |
| ——— kidneys | 3 | |
| ——— ovaries | 2 | |
| ——— uterus | 4 | |
| | — | 135 |
| | | 203 |

"In the other fifty-six corpses there was no visible evidence of disease in any of the viscera of the three great cavities! The spinal chord was examined in only two cases.

"From these dissections it follows: 1. That lesions of the brain, the organ of the intellectual functions, are in the proportion of one to two of those of the other viscera; 2. That more than *one* in *five* corpses of maniacs present no evidence of any disease whatever! 3. That in a great majority of cases, the insanity was a sympathetic affection; and, 4. That as, in more than a fifth of 259 dissections, no lesion or alteration could be detected, it strongly corroborates the opinion, that, when such lesions or alterations are observed, they are posterior, and not anterior, to the development of the mental derangement." 75.

Certainly they are posterior. Take the pleura, the peritoneum, the stomach, the liver, or any other organ. The disorder of function always precedes the change of structure. In the brain this is peculiarly the case, from the multiplicity and spiritual nature of its functions. But, as our author observes:—

"From whatever predisponent cause insanity may proceed, if it be not primarily an organic affection of the brain, it ends in being so. This seems demonstrated by the facility of its cure at the beginning of an attack of mania, comparatively with the attempt made at a more protracted period." 76.

Although absurd speculations have been entertained respecting the character of the living, as ascertained by the appearances of the dead brain; yet one thing is certain, that—"in the brains of connate fools, some defect or anomaly is *always* discovered, from which it might be inferred, that for the mind to be perfect, the organ of the mind, *ab origine*, must also be perfect." This is a natural enough conclusion—and although men have appeared to be possessed of *perfect minds*, whose brains were found imperfect after death, yet it by no means follows that the cerebral fault occasioned no corresponding defect in some of the mental faculties. Thousands, nay millions of men are going about, in the enjoyment of complete sanity of mind *apparently*, who are nevertheless, as decided MONOMANIACS as ever entered the walls of Bedlam. We need not travel out of our own profession for examples. There are maniacs writing in our medical journals—maniacs lecturing in our public theatres—maniacs editing the productions of maniacs—and maniacs reading and listening to the effusions of madmen! The senate, the pulpit, the bar, offer similar examples.

Dr. Burrows enters into a slight criticism on the doctrines of some of the Continental pathologists, who think they have discovered a certain correspondence between organization and insanity. Thus M. Bayle maintains that mental derangement, in a majority of cases, is the result of positive chronic inflammation of the membranes of the brain.

"He describes two kinds of inflammation, each having perfectly distinct anatomical characters and symptoms; the former he denominates chronic or latent *arachnitis*, because it principally has its seat in the arachnoid; and the latter, *chronic meningitis*, because it conjointly affects the pia mater as well as the arachnoid membrane. The latter affection, he asserts, is so uniformly attended with incomplete paralysis, that of 1453 cases of mental alienation, a fifteenth of the men, and a twenty-eighth of the women, were affected with this symptom." 78.

This author is confident that he is able, not only to connect specific symptoms of mental disorder with specific morbid conditions of the encephalon, but also to shew that, in a great proportion of cases, the commencement of the mental disturbance is to be imputed to a chronic disease of the membranes, of which he describes the forms, stages, and complications.

"He concludes, that the kind of mental alienation he has described is the effect of the irritation or inflammation of the gray substance of the brain, which immediately deranges its functions. This irritation and this inflammation are in their turn direct results of a chronic inflammation of the membranes, which commences on their internal or cerebral face.

"Of one hundred dissections of the brain, he did not meet with a single exception, he says, to the diseased appearances which are connected with the symptoms of this form of mental disorder." 80.

CALMEIL has pursued a similar route, and drew his observations from the same public establishment—the CHARENTON.* There are some discrepancies of opinion, and even of facts, between these two authors—and though—"it is impossible not to suspect that too much enthusiasm and aptitude for theory have influenced the pursuit—and that extraordinary facilities for morbid examinations sometimes tend more to encourage speculative theories than to advance truth," yet we cannot but lament that the *overflowings* of foreign zeal do not sometimes make their way into this country. Considering the strong tendency in the human mind to theorize, it is full as likely that those men should run into false doctrines who have no pathological data to direct them, as that men who are constantly comparing symptoms with dissections, should wander into the wilds of imagination. M. Bayle has been supported by Falret, as well as by M.M. Calmeil and Voisin, and our author confesses that—"though sceptical, he is bound to consider that the evidence is respectable." We need not dwell

* De la Paralysie considerée chez les Aliencs. 1826. Of which we have given an ample analysis in a former number.

on the opinions of many authors, as Vogel, Dumas, &c. &c. who considered insanity to depend on inflammation, chronic or acute, of the cerebral substance itself.

"I see no reason (says Dr. Burrows) why a state of irritation, or morbid action of the brain distinct from inflammation, should not obtain, and produce disorder of the intellectual faculties, as well as the more violent action of inflammation.

"Irritation, too, is not irrationally assumed to be the parent, or initiatory step to inflammation; for irritation primarily affects the balance of the circulation, and the impulse once given augments it, and induces re-action. Hence the nervous and vascular systems act and re-act on each other; and hence various diseases originate. Irritation, too, like inflammation, may have its varieties; for, in one part or texture, it may be excessive—in another, it may never arrive at activity—or either may exist in a part or organ in different degrees of activity." 85.

The examinations of the brain having proved unsatisfactory, pathologists have directed their attention to other viscera of the body, with the hope of being more successful. As in the brain, they discovered traces of disease in the heart, liver, stomach, uterus, &c. and thence drew their respective conclusions. Esquirol found a peculiar condition and position of the colon in many maniacs, and thought that this might operate as a sympathetic cause. But it has been found in men who had not been insane—and, in fact, it is rather a rare occurrence in those who have been so.

Dr. Burrows observes that, "the influence of sympathy in the production of insanity, is very extensive, and probably is the most common source of it." Van Helmont revived the ancient opinions respecting the sympathetic actions of diseased viscera on one another, and especially on the functions of the brain. He considered the viscera as the centre of sensations, whence they radiated on all surrounding parts. Bordeu, Barthez, Portal, Dumas, Cabanis, and most of the French physiologists, are imbued with the principles of Van Helmont.

"Although we know not the causes, nor the mode by which sympathies act, yet we have abundant proof of their operation in originating diseases which reciprocally act on the mind.

"There is no organ with the morbid actions of which the functions of the brain so frequently sympathise as the liver. As the connexion is intimate, so is it reciprocal; for morbid actions of the former equally, and perhaps as frequently, disturb the functions of the latter. In importance, the functions of this organ are only second to those of the brain, as far as regards the operations of health: and, as in the brain, so too in the liver, the circulation of the blood is complex, and very liable to be interrupted by extrinsic causes. Hence the greater facility of disturbing its functions.

"All the passions, anger especially, violently affecting the sensorium, act immediately on the liver; and every excess that disturbs the functions of the stomach, easily determines blood in undue proportion to the vena portarum, where, on account of the remoteness of this vessel from the heart, the motion of the blood is always sluggish, and therefore congestion is easily induced. The bile, consequently, is secreted in scanty quantities, the alimentary processes become ineffective, a morbid action of the connecting nerves follows, and the functions of the brain are implicated and disordered.

"Many facts attest, that blows on the head will create, not simply disordered function, but disorganisation of the liver; and *vice versâ*, nothing is more common than instances of mental disturbance originating in injuries of this organ, or in secretions of morbid bile, or obstructions of the biliary ducts by gall-stones, spasm, &c.

"Diseases of the hepatic system will even originate delirium, furious mania, melancholy, and suicide.

"Insanity is much more common among the lowest classes than the supporters of its mental origin are inclined to admit. Now, drunkenness is certainly the great vice of this class in Great Britain and Ireland, and the propensity is gratified usually by ardent spirits. In a table of 1370 lunatics, admitted into the Asylum at Cork, Dr. Hallaran says 160 were insane from this unhappy indulgence." 94.

The liver cannot be diseased from inebriation, without the stomach participating—and Dr. B. acknowledges that "a morbid condition of the chylopoietic viscera is sympathetically a frequent cause of mental derangement." But, after all, the brain must be affected before insanity can take place. Affections of other organs, like moral emotions, may be the remote causes—the proximate cause must be in the organ of the mind.

### COMMENTARY IV.—HEREDITARY PREDISPOSITION.

This chapter or commentary may be dispatched in a few lines. No pathologist now entertains doubt or cavil respecting the transmission of a disposition, predisposition, (or whatever other name we may choose to give it) to certain diseases, from parent to progeny. This truth applies equally to mania as to phthisis—another proof of the corporeal nature of the malady. A peculiar organization of the brain predisposes to insanity, in the same way as a peculiar organization of the lungs predisposes to consumption! The features of internal organs are transmitted like the features of the face. The hereditary qualities of the mind are only marks of hereditary organization. We shall quote one short passage from this commentary.

"My opinion upon two points relating to this interesting question has been sometimes professionally required by those contemplating marriage, and who were conscious that insanity had existed in one or the other of their progenitors; First, whether a person born of parents in whom insanity has never been developed, but who, one or the other, were descended from a family so afflicted, was capable of propagating it in his own children? Secondly, whether a child born before insanity had been developed in either parent was as liable to become insane as one born after it had been developed?

"To the first question I have answered in the affirmative; because I have met with many insane persons neither of whose parents had themselves been insane, but the progenitors, brother, or sister, of one or the other of those parents, were so.

"To the second I have replied, that a child born either before, or after the accession of insanity in a parent, provided that parent's progenitors or relations in blood had been insane, was liable to hereditary insanity. But if the insanity of the parent were adventitious, and not hereditary, the child born before the mental disorder had occurred of course could not have it by inheritance; but how far a child born after the occurrence of the adventitious insanity was liable, I could not decide." 107.

We must pass over the remaining commentaries in the first part of the work, in order to direct as much as possible of our attention to the second part.

### PART THE SECOND.

This part opens with a long examination of the definitions and divisions of insanity—the former of which is considered an "ignis fatuus in medical philosophy, which eludes and bewilders pursuit." Of the multitude of terms which have been invented for the disease Dr. Burrows prefers INSANIA, a name sufficiently recognized and sanctioned by ancient and modern authority, and which, he thinks, may fairly comprise "every form of intellectual disorder."

"All the causes, physical and moral, of insanity have existed since the origin of man. Hence we may conclude, that all those forms which the moderns have arranged as so many genera or species, were familiar to the ancients, who, above all, were most exact in clinical observation and accurate delineation of symptoms. They were content with simply dividing all mental disorders into two principal affections, mania and melancholia, to which they added frenzy or fury." 250.

Surely Dr. B. must have been precipitate in concluding that the progress of civilization has not multiplied and added greatly to the catalogue of moral and physical causes, not merely of insanity but of many other diseases.

"Dissections demonstrate that the morbid appearances in mania and melancholia are the same, and the like in respect to all the varieties recognised; such as monomania, theomania, demonomania, erotomania, suicide, lycanthropia, zoanthropia, panaphobia, nostalgia, &c. No form of insanity is characterised by any peculiar organic change. Such investigations, therefore, oppose all divisions founded on organic causes, though they confirm most satisfactorily the common origin and relationship of every form which presents itself." 251.

If no form of insanity be characterised by any peculiar organic change, how can it have been demonstrated that the appearances in all the varieties are the same? There is, indeed, considerable looseness as well as confusion of thought—or at least of language, in many parts of this work, as if it had been written in haste, though its composition occupied a period nearly as long as that of the siege of Troy. While our author pronounces all classification of mental disorders as "worse than useless," yet he admits that "a distinction between them (mania and melancholia) should be recognized and preserved," not only for convenience, but because the treatment applicable to the one is not always so to the other. Upon the whole, Dr. Burrows considers Dr. Esquirol's division into four species, *mania*, *monomania*, *demency*, *and idiocy*, as the best, though he objects to the term MONOMANIA, as a substitution for melancholia. His principal objection hinges on the ORGANOLOGY of the phrenologists—a science which seems to haunt the worthy Doctor like a dæmon, disturbing his repose, and exciting his ire or ridicule, on convenient or inconvenient occasions. By a most singular mode of reasoning Dr. B. concludes, that as the number of *hallucinations* is infinite, while that of the organs is limited, so the delusion "stamped on a monomaniac's mind (which differs in every case) cannot be the emanation of a distinct organ or portion." Granting for a moment, that there was an organ of RELIGION, might not there be many *forms of hallucination* dependent on the deranged function or structure of that organ; without any great violence to probability or analogy. If one man paid his orisons to the devil and another to our Saviour, in an extravagant or insane degree—or if one man prostrated himself before each cow that he met, while another watched eagerly for the rising sun, as the god of his idolatry,—would not Dr. B. consider all these individuals as affected with religious monomania, though the hallucinations were as different as day is from night?* We cannot therefore see, with Dr. B.

* We would ask Dr. B. whether a diordered stomach will not, at different times, produce different symptoms, even in the same individual; and yet no one would argue from this that each train of symptoms must have a separate stomach for its source. It may be the same with any of the organs composing that congeries or aggregate called the brain.

that, "there is, at present, great danger in using the word monomania," lest it should be "applied in one sense, while another is meant." The Doctor, however, prefers the term MELANCHOLIA, and as long as we know that by this he means MONOMANIA, why let him have his way. For our own parts, we think the latter term infinitely preferable, and that without any reference to phrenology. The order which Dr. B. adopts runs thus: INSANITY—comprehending, 1. Delirium—Delirium Tremens—2. Mania—Puerperal Insanity—3. Melancholia—Suicide—4. Hypochondriasis—5. Demency—6. Idiocy.

The Second Commentary of this part of the work is very long and desultory, though abounding in curious and instructive facts and observations. It is on the "CHARACTER of INSANITY"—and we can only notice some of the phenomena grouped by the learned author under the heads—physiognomy, position, sensation, muscular powers, fasting, odour.

1. *Physiognomy.* All men, and even many animals, are physiognomists. But if it be difficult to judge, from the expression of a sane person's features, of what is passing in the mind, how much more difficult must it be to form a correct judgment from his whose aim is to deceive—whose mind is regulated by no rule, and whose ideas and actions are wild and incoherent?

"The feature which undergoes the most singular and striking change is the eye. The pencil or graver may portray, with tolerable fidelity, the contour of the countenance in the varied states of maniacal fury, melancholy, fatuity, or idiotism; but the skill of art is vain in delineating the peculiarities in the eye of the lunatic. The pen is equally, or perhaps more, incapable of describing it. Now red and fierce, then audacious, threatening, steady, or mobile; sometimes brilliant, quick, and flashing fire; or dull, desponding, fixed, and vacant; and in most cases presenting a remarkable glassy or shining appearance." 282.

Those who are familiar with the insane can seldom be mistaken, when they pay attention to the eye. Language and behaviour may deceive;—the mobility of feature may be rapid as the imagination is vivid; but when every feature shall vary, or be kept under control, the eye may still indicate the erring thought.

The suicidal tendency, Dr. B. observes, is denoted by a peculiar expression of the eye, which cannot escape the practised attendant.

This organ indicates the material condition of the brain, as well as the state of its intellectual functions. Phrenitis, hydrocephalus, apoplexy, epilepsy, as well as mania, have their appropriate expression of eye.

"Sometimes the pupil will be dilated to an extreme, and the patient will bear, without blinking, the direct rays of a vertical sun; sometimes the pupil is concentrated to a pin's point, and light is a source of extreme irritation. The eye is frequently much protruded in mania; but this is not owing to extreme pain, as in phrenitis, or to greater activity or fulness of the blood-vessels, as may be suspected in hydrocephalus or apoplexy. During the maniacal paroxysm, as when horror-struck, the eyelids are forcibly separated and retracted, so as to expose a circle of the surrounding albugineous substance, which gives a greater appearance of prominence to the orb than is really the case. Sometimes the eye-lids recede, from absorption of the adipose supporting the eye-ball, and general emaciation of the face, as is seen in persons after extenuating illness; and then also the eye looks enlarged and protuberant." 283.

It is curious that, in many persons predisposed to violent mania, the iris is so black that it can scarcely be distinguished from the pupil. The melancholic have generally blue or gray eyes. Esquirol has given graphic sketches of the principal forms of insanity—and Dr. Morrison has published in his Outlines several excellent portraits.

2. *Position.* Maniacs and young children are often incapable of indicating the actual seat of pain, and it can only be discovered by external signs, and especially attitudes. Ease and freedom of position, with soft and regular breathing, indicate health—supination denotes great prostration of strength—"lying on the belly is a sign of pain in that part, and is often a prelude of delirium." Many lunatics rub or press their heads with their hands, and acknowledge that they feel pain there, if interrogated. Maniacs and melancholics, have often a decided partiality for the sitting position—many others crouching with their knees folded towards their chins. Not a few have a positive horror of the recumbent posture—a phenomenon which seems to indicate a determination of blood to the brain in that position. Maniacs, however, assume various positions, many of them apparently connected with the mental hallucination that reigns in the imagination. Thus a religious lunatic will take a fixed posture, directing his eyes towards the Heavens, with steadfast look as if lost in adoration. Sometimes he bursts into immoderate laughter at his own conceits:—while others are immoveable—fall into imperturbable silence, with averted eyes, apparently overwhelmed in concentrated sorrow. Such movements and actions, as Dr. B. justly observes, are the offspring of a creative and bewildered imagination, and are not to be regarded as indications of peculiar corporeal feelings.

"3. *Sensation.*—Some authors, and Darwin among them, have supposed insanity to be a disease of pain; and hence the cries, screams, moans, howls, and vociferations of the insane, have been ascribed to the bodily agony they suffer. We must not, however, imagine that these are indications of bodily suffering, any more than that they really feel pain in the various remote parts to which maniacs often refer. When cephalalgia is complained of, it is generally as a precursory symptom, or in the incipient stage of insanity; but the existence of it is never expressed by cries, &c. Severe pains have been felt in the stomach, intestines, and other organs, previously to a maniacal paroxysm; but when the delirium of insanity is developed, the morbid action of the remote part being transferred to the brain, the organ originally affected is commonly relieved from suffering." 286.

We do not quite agree with Dr. B. in the foregoing passage. He seems to overlook the sensations of organic life—in other words, the sensations of the ganglionic nerves, which cause the most horrible sufferings without any thing like common pain, as experienced in the nerves of relation, or cerebro-spinal nerves. We do not wonder, therefore, that, on enquiry, Dr. B. has not been able to elicit from recovered lunatics any information respecting the cause of those "screams, moans, and howls" which they had formerly uttered. The two following cases are curious, and physiologically interesting.

"A gentleman, aged thirty-six, insane, with a strong hereditary predisposition to suicide, contrived, during the temporary absence of his keeper though his legs were fastened together, to kick a hole in the fire-guard, and thrust his feet into a quick fire, which he made more fierce by tearing up a book, and thrusting the leaves in. He was found a few minutes after, sitting very composedly in this position. His toes, and part of one foot, were severely burnt; the other escaped with a smart scorching. In the burnt foot, inflammation, extensive and deep eschars, and mortification, with sloughing of the muscles and tendons, followed; and, finally, all the bones of the toes, and some of the metatarsal bones, sloughed away. The cure of this foot occupied more than a year; the scorched one soon got well. But neither during the combustion of the toes, nor for months afterwards, upon removing the diseased parts, or dressing the wound, was any pain expressed. But when the mind improved, and the desire of suicide diminished, which it did long before the wound healed, he complained violently of the pain he suffered from it, or when it was dressed.

"A French dragoon became insane from a *coup de soleil* during the Spanish campaign. In his delirium he found means to get at a vessel on the fire filled with boiling water, of which he drank, at a draught, about a pint, and then quietly returned to his bed. He remained two days without eating or drinking, and without complaint, though his mouth was much inflamed and eschars had formed. Six days after this circumstance, an abundant ptyalism came on, which was succeeded by a copious diarrhœa; and in three or four days afterwards he recovered his health and intellects." 290.

4. *Muscular Force.* This, in the insane, is sometimes truly marvellous—and is evidently dependent on the violent excitation of the sensorium. We all know what power even a delicate female will exert, in a paroxysm of hysteria. The following passage is worthy of the attention of the junior practitioner.

"The astonishing muscular power exercised by the insane is often mistaken for proof of real strength; and hence a depleting practice is often adopted, fatally injurious to the patient. The fury which prompts this violent exertion may be abated, or even subdued, by these means. But it should ever be remembered, that a state of exhaustion naturally follows these paroxysms; and then the vital power, which the depletory measures have subtracted, may be wanted to prevent worse consequences." 293.

5. *Fasting.* The insane not unfrequently resist the natural desire for food, either from a suicidal tendency, a fanatical resolution, or a dread of poison. In these cases, the constitution will suffer and sink, if nutriment be not introduced by force or persuasion.

"Nothing is more common than for the insane to object to food from an apprehension of poison in it; but it must not be always considered as a delusion when they refuse it on that ground; for sometimes their taste is so perverted or depraved, *that all substances partake of the same flavour, and often a very nauseous one.* We must, therefore, if possible, ascertain whether the rejection proceed from indifference, religious scruples, a determination to starve themselves, fear of poison, or real distaste; because the mode of acting must accordingly vary." 296.

The explanation of Dr. B. as marked in Italics, differs from that of some of our Continental pathologists, who attribute the horror of food to an inflammatory state of the gastro-intestinal mucous membrane.

*Odour.* Mania is distinguished by a peculiar odour, which can never be forgotten, after being once felt. "It is not," says Dr. B. "the *hircum olet* of Horace, but is a smell quite unique." It is not always an attendant on mania; but when present, Dr. B. considers it "a pathognomonic symptom so unerring, that if he detected it in any person, he should not hesitate to pronounce him insane, even though he had no other proof of it." This is strong language, too strong, perhaps, for medicine.

"I remember the case of a very delicate young lady, of good family, and highly educated, who became insane; but whose family would not admit the correctness of their physician's judgment, till her mother, having somewhere heard of this characteristic symptom, upon entering her daughter's chamber before she had risen, detected this peculiar fetor; and then she yielded to conviction of the nature of the malady." 297.

This young lady was the patient of the writer of this article; and when the maniacal paroxysm was strong, and her temper much irritated, the odour was almost insupportable.

The second Commentary, in this division of the work, treats of DELIRIUM, chiefly with the view of drawing a distinction between this affection and Insanity. The distinction is more easily made in practice than in words, which is so far fortunate, and therefore we shall pass it over. The commentary in question, however, contains a great deal of research and information respecting the various kinds of delirium, as that of acute diseases,—as resulting from wounds—from long fasting—and from the approach of death:—

> "'Expression's last receding ray,
> A gilded halo hovering round decay;'"
>
> "The simple but sublime elevation of the mental over the corporeal essence, when the present world and all its attachments are unloosed—when we cast off all the grossness of mortality, and are putting on immortality." 317.

We cannot but look upon the above specimen of delirium,—"that state of the intellectual faculties *often* evinced at that awful crisis when death approaches, and which imparts to the words and acts of the dying an *apparent spirit of divination*," as an amiable flourish of the worthy Doctor's own imagination. From circumstances unnecessary to be mentioned here, we have had but too many opportunities of witnessing the dying scenes of humanity. We are sorry to say that we never observed any of these "gilded halos" hovering round the couch of death, and marking the dissolution of that mysterious tie which holds mind and matter united in this world. On the contrary, it has but too generally happened that the mental manifestations sank, *pari passu*, with the bodily functions—nor did we ever see an exception to this rule, where the material organ of the mind was involved in disease. Small then is the hope of immortality, as founded on these "words and acts of the dying," on the "apparent spirit of divination," which is said to be "so often evinced at that awful crisis when death approaches."

The fourth Commentary is on DELIRIUM TREMENS, a disease which has obtained so much attention in this Journal, of late, that we need not dwell on the subject in this article. We shall, however, offer one or two short extracts indicating Dr. B.'s pathology and treatment of the disease.

> "The nervous system is, by frequent application of so strong a stimulus, at first violently excited and irritated; and by repetition, the sensorium is so affected as to occasion a temporary effect similar to partial palsy. In time, organic changes of the viscera implicated in these morbid actions take place, and death, or permanent alienation of mind, ensues." 326.

### TREATMENT OF DELIRIUM TREMENS.

> "But the first and most essential duty, upon being called to a patient with delirium tremens, is to ascertain to what extent his previous habits of drinking have been carried, and how far his constitution has suffered, and to prescribe accordingly. If, as I have said, the constitution be little impaired, and the symptoms of cerebral excitation run high, very moderate depletion by cupping, and purging, and the antiphlogistic diet, may be premised; and the exhibition of opium, in such doses as will induce sleep, should follow. If, on the contrary, the patient is advanced in years, and is a confirmed bibber, abstraction of blood should be avoided; his bowels should be merely kept soluble; a little spirit or wine, diluted with water, and light nourishment be allowed; and he will require larger doses of opium before sleep can be produced.

"Even if opium do not procure sleep, yet there is this decided advantage from its exhibition—a state of quietude is obtained, equally desirable for the patient and his attendants." 333.

We must pass over the remaining Commentaries in the second part, containing dissertations on puerperal insanity, on senile insanity, suicide, hypochondriasis, demency, and idiocy, because we wish to dedicate the sequel of this article to the treatment of the malady. This subject is commenced in the first commentary of part V. and is continued through five commentaries.

Our author wisely disclaims all knowledge of "an antimaniacal remedy," nor does he profess the charm of *novelty* in his treatment of the disease. He tells us that the plans laid down by Areteus, Celsus, Galen, &c. although founded on a different pathology, are, "in most points, well adapted to the pathology founded on the anatomical discoveries of modern investigations." Dr. B. assures us that he has not attempted to defraud the moderns of what is owing to them; but he acknowledges that he is more indebted to the ancients than the moderns for what success may have attended his efforts. Dr. B. observes, and truly too, that—"no mental disorder can originate except through corporeal disorder; and the *only* remedies for a mind deranged are those which apply to the corporeal derangement that *influences* (why not say, at once, *causes*) the mental derangement." The physic for the mind is moral discipline. The cure of insanity is usually divided into medical and moral. Dr. B. begins with the *medical*—we shall take the liberty of reversing the order. We shall begin with the subject of

### SECLUSION.

There is no general maxim in the treatment of insanity, wherein medical practitioners are so unanimous, as that of separating the patient from all customary associations—from family—from home. We do not much wonder that the feeling of the non-professional public should be strongly opposed to the faculty on this point. Dr. B. has seen a few, and but a few, patients recover in their own domiciles.

"The only case where it (separation) may be dispensed with, is when the affections are in no way perverted, nor the existing delusions associated with home, or any person or object about it. But even then there are other circumstances to be considered which may prevent recovery while in that situation.

"Few persons when they become insane acknowledge being so: consequently, when they find themselves placed under control in their own houses, denied intercourse with their families, and their orders not only disobeyed, but their own servants concurring in controlling them,—they are naturally infuriated to a high degree, or imbibe a plausible and strong suspicion that a conspiracy is formed against their life, liberty, or property. These are irresistible reasons why insane persons should be removed from home." 697.

On the other hand, where the insane is removed to a strange place, and put under the control of strangers, a moral influence is thereby established, to which he usually submits, and a cure is to be expected which could never take place at home.

### MORAL TREATMENT.

Dr. B. acknowledges that the moderns are superior to the ancients on this point. This is curious too, if "the qualifications (of the physician for moral management of the insane) are intuitive, not acquired." Here then is another link between poetry and madness. POETA NASCITUR NON FIT. So it is, we find, with the MAD DOCTOR! But though specific rules cannot be laid down for the management of the insane, "yet a few general principles are recognised which embrace almost the essence of this department." These are:—

"First, Never to exercise the mind of an insane person in the sense of his delirium.

"Second, Never to openly oppose the morbid ideas, affections, or inclinations of the insane.

"Third, which is a consequence of the two preceding, to give rise, by diversity of impressions, to new ideas and feelings; and thus, by exciting fresh moral emotions, revive the dormant faculties.

"Fourth, Never to commit one's self to an insane person by a promise; but if inadvertently a promise be given, faithfully to adhere to it, unless certain that the fulfilment will be attended with worse consequences than the breach of it.

These principles are not for the government solely of the physician, but of every one who has the charge, or is attending on, or visits casually, a lunatic." 668.

It is in the stage of convalescence that the great art of moral management becomes conspicuous. But as this art cannot be communicated in words, we shall dwell no longer on the subject.

### RELIGIOUS COMMUNICATION.

This is a mooted point in the present godly times. If religion be sometimes the cause of insanity, as Dr. B. himself allows, it may easily be conceived that the application of the same, in the way of cure, must be a very delicate operation, and require unusual discretion. This application, Dr. B. avers, and we believe, with reason, can never be admitted as a general principle. Divine service, in the usual forms, has been tried in Bethlem, Glasgow, Lancaster, and some other asylums, and it is said, with advantage, when the discourses were suitable to the patients selected. But the statements on this head are to be taken, cum grano salis, for obvious reasons. We are not certain that Dr. B. was not influenced a little by the opinions of the PURITANS, rather than by his own experience, when he penned the following sentence.

"Governed by these rules, (judicious selection and suitable discourses) I have never experienced any ill, but, on the contrary, much good effect, by a proper attention to religious observances among the patients of my own establishment." 680.

We should be glad to know who it was, Dr. Burrows, or the Parson, who was constituted the judge of the suitableness of the sermons? It could not surely be the latter; for how was he to know the state of the patients, or their capacity for religious discourses. It must have been a novel sight to observe the physician in consultation with the minister, every saturday night, on the nature of the sermon which was to be preached next morning!

"Before religious instruction in any form be attempted, let it be received as a maxim, that an intimate knowledge of every patient's state of mind, and of his former and present opinions regarding religion, should be first ascertained; and till all doubt on this head be removed, every interference should be suspended. This information, among a great number, is difficult to obtain. Even when obtained, the admissibility of this powerful auxiliary, in assuaging the anguish of a troubled mind, and aiding the recovery of convalescent lunatics, must be left to the judgment of the physician; but, above all, if in an asylum, to the accuracy of the superintendent's discriminating faculties. Hence it is easy to conceive how great an adept he ought to be in fathoming the recesses of the human mind." 681.

We apprehend the foregoing passage goes far to settle the question of preaching to the insane. But we must now descend from a higher to a lower flight —from religion to physic.

### MEDICAL TREATMENT.

This department of the work before us, and indeed of every English medical work, is highly characteristic of the national polypharmic feeling. A Continental physician would luxuriate in the delineations of the indications to be pursued, and the pathological conditions which were to be obviated, leaving, almost entirely, the means for fulfilling both these intentions to the judgment of the practitioner. Not so the English physician. He displays an almost interminable list of individual medicaments, descanting on their properties, doses, virtues, vices, &c. &c. till the inexperienced is completely bewildered as to the choice of so many remedies for one disease! Thus the third commentary of the fifth part of Dr. B.'s work presents us, under 21 heads, with a most delicious bill of fare, in the treatment of insanity; the very sight of which might go far to cure a madman—or make a sane man mad.

"Abstraction of Blood, General and Topical—Dry-Cupping—Refrigeration—Gyration and Swinging—Sleep—Narcotics—Blistering—Setons and Issues—Artificial Eruptions—Bathing—Purging—Vomiting—Nausea—Salivation—Digitalis—Prussic Acid—Camphor—Spirit of Turpentine—Tonics—Tobacco—Diet." xvi.

We do not quote this bill of fare with any intention of reflecting on Dr. Burrows. Though the plan may appear less scientific, it may perhaps be more useful than that adopted on the Continent.

Dr. B. remounts, of course, to the cure of King Proteus's daughter, by means of PURGATION—a remedy which has not lost in reputation or repetition since that period. Our author wisely advises us to make inquiry into the cause, nature, and seat of the malady, before we begin our methodus medendi—and if we cannot discover these, to wait a few days, where no symptom threatens, keeping the patient separate and quiet—and merely attending to the bowels. Dr. B. thinks that "the pathological division of insanity into the different *stages*, has done more to advance the treatment of the malady, than even the clinical experience of the ancients, or the *morbid discoveries* of modern anatomists."

"The conviction, however, has at length arrived, that insanity is a purely corporeal disease, and, like other corporeal diseases, is amenable to medical skill.

"In every case of insanity there is a diseased action going on, and each demands a separate examination: the features may be there, but be as varied as the expression of the human countenance. Hence none but general principles can be laid down, nor is any systematic treatment admissible." 577.

Dr. B. further observes, that the several stages which insanity pursues in its course, "testify that the brain, the organ of the mind, assumes different mor-

C 2

bid conditions—first functional, and then structural—functional in the first three stages—structural or organic in the last." This pathological view is to be our guide in therapeutics.

"In the *incipient stage*, there is evidence of great vascular excitation and cerebral irritation, and this stage must be met by a correspondent treatment. Here are indicated repeated topical abstractions of blood from the head or contiguous to it, shaving the head and refrigeration, so long as there is preternatural heat of the scalp, cautious general blood-letting, even in the plethoric and robust, very moderate in the delicate, though young, purging, vomiting after the vessels of the head are unloaded and the bowels evacuated, nauseating doses of tartarised antimony to moderate the circulation and excessive violence, the digitalis in gradually augmenting doses, till the pulse intimates reducing the dose, saline draughts, and moderate diet.

"In the *active* or *confirmed stage*, the fury and violence of mania, or the despair of melancholia, with their concomitant mental delusions, may persist, yet the symptoms of physical excitation attending the incipient stage subside or intermit, and occasionally only return.

"When the symptoms of excitation recur, they must be treated as in the first instance, except that neither depletion by local or general bleeding, nor by any evacuants, should be so active or copious. The system will not in this stage bear them so well; on the contrary, light tonics and the shower-bath are of great use, even when moderate topical bleeding and purging are indicated; and when the exacerbation of a paroxysm ceases, more powerful tonics, as chalybeates, cinchona, cold-bathing, and a better diet, are admissible. It should also be observed, that in melancholia the class of remedies which are designated anti-nervines, are useful adjuvants.

"In the *convalescent stage*, if symptoms still denote cerebral congestion, gastric irritation or uneasiness, or intestinal irregularities, they should be attended to until they are removed. In this stage, moral treatment besides is especially indicated." 581.

We have now to glance at some of the principal items in the therapeutic catalogue.

1. *General Bleeding*. This is a measure which has been largely employed, up to the present period. We agree with Dr. B. in doubting its efficacy, except in very restricted cases. Indeed we have no doubt that copious abstractions of blood are generally fraught with mischief.

"Following example rather than experience, I tried depletion by blood-letting for several years; but discovering my error, I became more cautious; and, I believe, that I have scarcely ordered venesection in six cases of simple mania or melancholia in as many years. My conclusion is, that since I changed my practice, more have recovered, and certainly the cases have been less tedious and intractable." 583.

2. *Local Bleeding*. This is far more eligible. The primary symptoms, both in mania and melancholia, indicate increased activity in the circulation of the brain.

"The partial pains, tension, or throbbing in the head; extraordinary heat of the scalp, flushed face, blood-shot or glistening eyes, and general confusion of ideas, mark cerebral determination or congestion." 589.

Dr. B. does not recollect a single exception to the utility of local depletion, in cases of recent insanity. Cupping or leeches are the modes to be employed —and our author prefers "cupping on the occiput," to all other modes. Leeches are the best substitute, where there is a horror of cupping. The quantity of blood to be abstracted, and the repetition of the operation, must be left to the judgment of the practitioner.

"In cases of nymphomania, all the distressing symptoms whence this affection derives its name have been removed by the application of leeches to the vulva. In like manner, improvement of the mental faculties, dependant on menstrual obstruction, follows cupping on the sacrum. Sympathetic delirium from an affection of the liver, has subsided by local abstraction of blood from the hepatic region." 592.

Dr. B. has found sensible advantage from dry cupping, in cases where emaciation and debility positively forbade the detraction of blood.

3. *Refrigeration.* Shaving the head, and keeping it covered with refrigerating evaporating lotions, are universally admitted to be most important measures. They almost invariably produce a calming and even soporific effect, in violent mania. Equal parts of spirit, vinegar, and water, form a very good evaporating lotion.

4. *Gyration and Swinging.* These were strongly recommended by Darwin, Cox, Hallaran, Horn, and others. But some fatal accidents having taken place, the employment of these machines is far from general.

"It is described as seldom failing to produce copious evacuations in the most obstinate cases, provided that, on increasing the velocity of the swing, the motion be suddenly reversed every six or eight minutes, pausing occasionally, and stopping its circulation suddenly: the consequence is, an instant discharge of the contents of the stomach, bowels, and bladder, in quick succession. Should the stomach only be acted upon, a purge should be afterwards given." 601.

Dr. B. has, with many others, been deterred from introducing it into his asylum, on account of the popular prejudice engendered by the Parliamentary Committee of 1815-16.

5. *Sleep.* Dr. B. thinks there is commonly by far too great a solicitude among practitioners to procure sleep for the insane.

"A maniac awoke from sleep artificially obtained, is a giant refreshed. New activity is imparted to the sensorium, and his muscular powers are recruited. If he have lost by it one hallucination, another assumes its place, more wild, perhaps, and extravagant than the former, and his waking dreams are the more vivid; hence his violence and raving are increased, and the power of continuing them prolonged." 607.

In the incipient stages of insanity, there is too much excitement—in the confirmed and incurable, too little, though sometimes accompanied with great irritability. Whatever diminishes the excitement, in the former case, as local depletion, cold, &c. will induce sleep. In the latter case, the opposite plan, under judicious inspection, may be often serviceable in procuring repose.

6. *Narcotics.* These remedies have produced great discussion among physicians.

"Maniacs will generally bear large quantities of opium and other sedatives better than they will support remedies which weaken the vital powers. But opium, when the excitation is great in a full and strong habit aggravates; when the excitation is moderated by previous depletion, or the habit is reduced by long continued mania, stimulants, like opium, wine, porter, &c., tranquillise and prove soporifics." 610.

In the early stage, the vessels of the brain should, of course, be unloaded before narcotics be ventured on. When given, they should be in large doses, or none at all.

"I have never ventured beyond five grains of purified opium as the first dose. In those cases where I have deemed an anodyne admissible, I generally begin with three grains, and repeat one every two or three hours. I have never in this way exceeded twelve grains; and if sleep has not then followed, I have desisted." 613.

On the effects of hyosciamus niger, blistering, setons, and tartar-emetic frictions, we need not dwell. Dr. B. appears to have been greatly disappointed in the expectations he had formed of the latter remedy, in consequence of the representations of Jenner and others. We must always be prepared for disappointments when the favourite nostrum of a medical writer is submitted to the test of experience by others.

7. *Bathing.* This is a subject which has given rise to innumerable discussions in ancient as well as in modern times. The following are Dr. B's opinions respecting cold and hot baths, together with their modus agendi.

"The effect of general bathing, whether warm or cold, is imitative of the process of fever, which, as I have shewn, will suspend the maniacal action, sometimes as long only as that lasts, and sometimes accomplish the perfect restoration of the intellectual faculties. Fever gives an impetus to the circulation, and distributes the blood through the encephalon with an activity that imparts new energies to the brain.

"Warm bathing immediately produces accelerated circulation; cold bathing mediately, by re-action. Hence, both are perturbators, and eventually may equalise the circulation, which, perhaps, in every case of insanity is, in one way or the other, disturbed. Consequently, provided the necessary precaution of evacuation be adopted in the plethoric, or those with a manifest determination of blood to the head, either warm or cold bathing may prove equally beneficial. In using the warm bath, the conjoint application of cold to the head may prevent the ill effects of determination, even when evacuation had not been premised; but the safer practice in such cases is, to prepare for its use by local bleeding and proper alvine dejections." 628.

8. *Purgation.* Black hellebore is the most ancient of all purgative substances, and has been celebrated in the cure of insanity, especially of melancholia, for 2000 years. So late as the days of Thomas Willis, twenty grains of calomel, the same of extract of black hellebore, and six grains of extract of jalap, were given for one dose, in insanity.

Drastic purgatives are sometimes necessary in the beginning of the disease, not only to overcome the torpidity of the bowels, but to clear away accumulations from the colon and rectum. When the evacuations have become natural, the bowels should not be irritated by powerful cathartics, but regulated, if possible, by diet, or by mild laxatives. There is a common but dangerous error prevailing, that the bowels of lunatics are peculiarly difficult to be acted on. This has led to very bad practice.

9. *Vomiting.* Of all measures for the cure of insanity, this is the most generally and strongly recommended.

"Evacuation, says the elder Monro, is the best cure, and vomiting preferable to all others; and if not carried beyond the patient's strength, nor crowded too fast upon him, his health of body will visibly improve so long as vomits are continued. The prodigious quantity of phlegm which accumulates, he observes, is not otherwise to be got rid of; and he adds, that purges do not operate so well till after vomits. Hallaran, however, advises, that purging should precede vomiting." 641.

Dr. B. conceives, and we believe justly, that the beneficial action of vomits in insanity, is not to be attributed simply to their evacuating properties, but rather to their well-known effects on the circulation generally. The following declaration of our author is not very consolatory in reference to emetics.

"Influenced, however, by the strenuous recommendations of emetics for the cure of insanity, I gave them a fair trial; and in several cases relied upon their operation together only with purging. I used in turn every substance in the materia medica possessing emetic properties, and marked with attention the effect of each; but I must conscientiously declare, that, after several years' perseverance, my confidence in emetics alone in cases of insanity has been entirely dissipated." 641.

Still Dr. B. has recourse to emetics occasionally; but only as in other diseases—"to free the stomach from troublesome ingesta, accumulated phlegm, or morbid bile."

"Doses of emetic tartar at such intervals as will keep up the nausea, rarely fail to reduce the most stubborn to subjection. Sleep, also, which in these cases is so desirable, will sometimes occur while in this state. This plan should be continued so long as it is positively useful, and no longer. I have known it pursued for a fortnight, and the hallucinations by degrees dispersed, or so weakened that the cure has been quickly accomplished." 643.

10. *Salivation.* Spontaneous ptyalism is a phenomenon often attendant on insanity, and probably led to the employment of mercurial salivation in this disease. Dr. B. has made many attempts to cure insanity by mercurial ptyalism, "yet he never succeeded but in one case to restore the mental functions by it." This was a case of melancholia.

11. *Digitalis.* This appears to be the most favourite remedy in mental derangement at present.

"I have never carried the dose beyond fifty drops of the tincture of digitalis of the London Pharmacopœia. Even in that quantity, by gradual increment, I have seen effects produced that have alarmed me for the safety of my patient; and therefore, if it has not answered in that dose, I have desisted from carrying it further, or suspended it altogether.

"Besides premising depletion, and purgation with calomel, Dr. Hallaran advises mercury to be given internally, so as to produce moderate salivation, as preparatory to the exhibition of the digitalis.

"Without any of the enthusiastic admiration and confidence in the virtues of the foxglove as an antimaniacal remedy which this respectable physician professes, I perfectly concur with him in considering it as having a very powerful influence, when properly administered, in all stages of insanity accompanied with great vascular excitement and a rapid pulse.

"I believe, also, that if the general rule he lays down, of previously depleting, and evacuating the bowels by calomel purges, be adhered to, the operation of digitalis will be found more uniform, not only in insanity, but in many active diseases where it now often proves ineffectual." 654.

The other medicinal remedies in the catalogue need not detain us any longer, and we have now, we hope, given a pretty full, though very general view of Dr. B's work. It is not one of those which are so diluted with words, that the valuable matter may be condensed into a nutshell. It is really a work containing an immense collection of important practical information from various sources, digested and commented on, by a man of sound judgment, accurate observation, and extensive experience. This may not appear a very high panegyric, but it is one which, we apprehend, will recommend the book more effectually to the attention of our readers, than whole pages of fulsome adulation.

